# The influence of skill-based policies on the immigrant selection process

**DOI:** 10.1007/s40888-022-00264-w

**Published:** 2022-04-04

**Authors:** Mariele Macaluso

**Affiliations:** Department of Legal Studies, University of Bologna (Italy), Bologna, Italy

**Keywords:** Immigration, Immigration economics, Selective policy, Selection, F22, K37, J24, J61

## Abstract

Understanding the type of immigration flow that maximises the expected economic benefits in the destination countries is one of the main debated topics both in the economic literature and in policy agendas worldwide. In recent years, governments have developed regulations of migration flows by adopting some form of selective immigration policy based on either human capital criteria or skill needs. Admission policies in the destination countries are likely to affect the direction and magnitude of selection as well as the socio-economic performance of immigrants. However, the relationship between quality-selective policy and immigrants’ skill composition remains largely unexplored. This paper aims to survey the existing literature on how selective-immigration policies shape the characteristics of immigrants from the receiving-country perspective. First, it introduces the main route of admissions and the theoretical models to understand how the direction of selection works; second, it discusses the theoretical models; third, it reviews the empirical works. A final concluding section briefly points out the actual findings and future avenues of work.

## Introduction

**Fig. 1:**
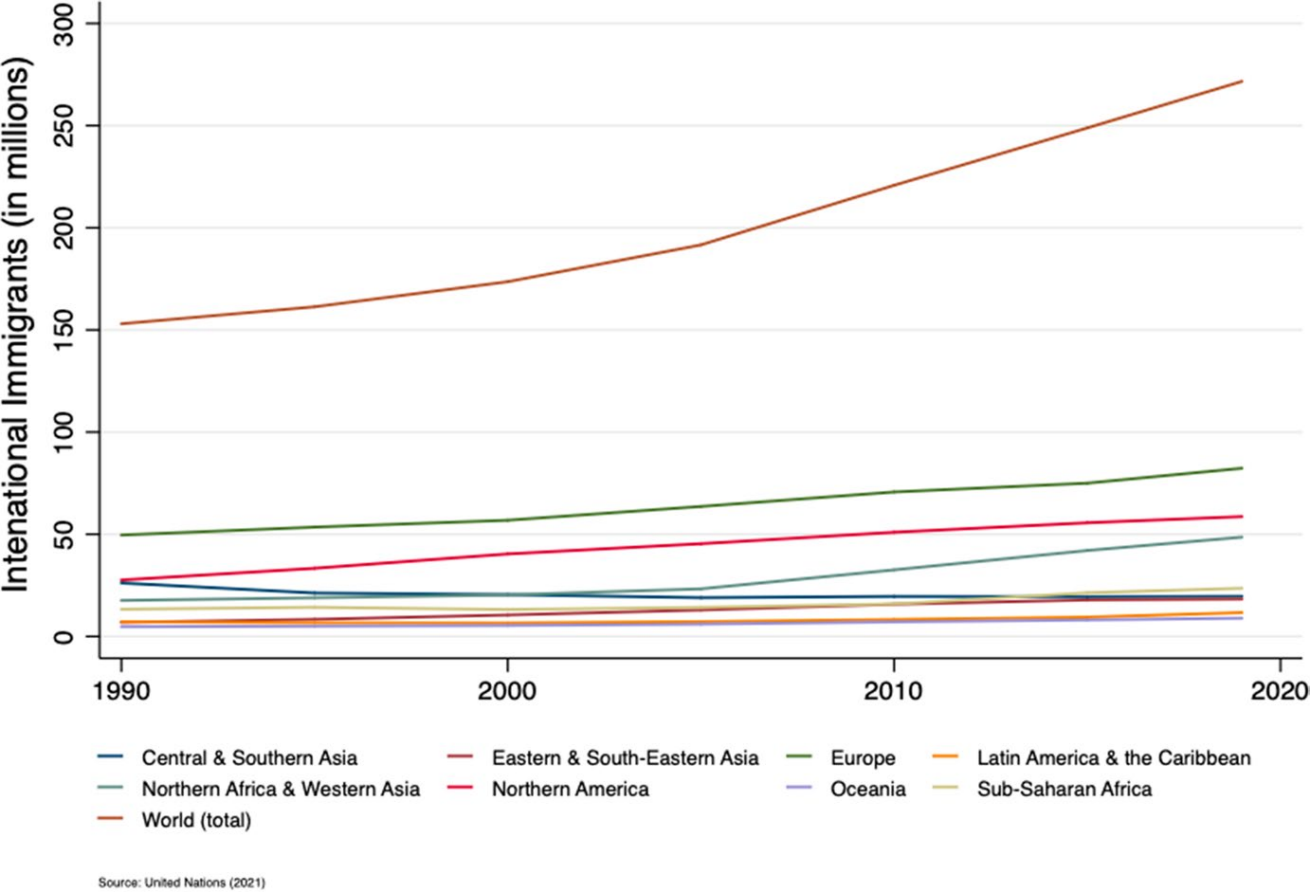
Number of international migrants by major area of destination

The importance of the nexus between policies and migration outcomes has gained increasing attention both in the economic literature and in global policy agendas. Concerns about the impact of immigration raise questions on what determines the size and the composition of immigrant inflows. Immigrants are indeed expected to positively contribute to economic growth by supplementing skills in relatively short supply and enhancing the labour force. Understanding the type of immigrants that maximises these expected economic benefits is one of the main debated topics. Figure 1 shows the total number of international migrants in 2019 reached nearly 272 million, following a stable upward trend (UnitedNations 2021).

The rise in international migration to developed countries has been marked by unprecedented institutional and technological changes, such as ageing, new skills needs and growing specialisation in human capital and digital-intensive activities. These current shifts have remarkably affected the migration patterns in terms of both demographic and skill trends. Between 2000 and 2016, the number of highly educated immigrants increased throughout the OECD countries by around 20 million (OECD, 2019). The demand for highly skilled labour has reflected the overall increase in education levels worldwide, while key sectors of many developed countries’ economies now suffer from specific skill shortages. In light of growing global competitiveness, many countries have selectively opened their labour markets to those applicants with higher levels of education or based on demand criteria while at the same time discouraging low-skilled migration. In this sense, institutional and regulatory contexts are crucial to whether migrants are eligible for a country and, if so, which destination they choose.

Economic literature has devoted attention to the selection and sorting of migrants across countries, considering migration as the result of spatial differences in the net returns to factor supply and the response to labour market disequilibrium (see Bodvarsson et al., 2015). As immigrants are not randomly selected from the population of origin country, literature has focused on understanding which subsample decides to move, such as the composition, or the characteristics, of foreign inflows. The policy is one of the determinants influencing the conditions of entry through specific immigration channels and the type of immigrants attracted to each destination. For this reason, the composition of immigrants is considered itself equilibrium outcome of these policies (Chassamboulli & Peri, 2020). Specifically, selective-immigration policies may act directly on selection by admitting only certain groups, but also indirectly by marking migration as costly for some immigrants than for others (Biavaschi & Elsner, 2013). In this sense, selective systems are considered a way to admit the highly educated and improve migrants’ quality. By “quality”, we understood a higher education level usually measured by the average log wage that migrants earn at destination (Borjas, 1987; Bertoli & Rapoport, 2015). Moreover, the quality of immigrants depends also on several characteristics, such as innate ability, talent, attitude towards risk, which remain unobserved for policymakers (Bertoli et al., 2016; Bertoli & Stillman, 2019).

This paper aims to survey the existing economic literature on how the adoption of skill-selective immigration policies shapes immigrants’ characteristics from the receiving-country perspective. On the other hand, this survey intends to foster future research on the possible consequences of a widespread shift towards selective immigration policy on the selection process. Despite the increasing importance of this topic, it is still unclear how immigration policy in the host countries is likely to affect the direction—positive or negative—and the magnitude of selection. The main reason is the lack of relevant data on both flows and policies that have severely constrained these analyses. In addition, few papers have investigated the impact of policies on current and future incentives as well as on other determinants of self-selection process—i.e. networks (Bertoli & Rapoport, 2015)—and other cultural and social makers, such as gender norms, family ties, ethnic enclaves, marriage and fertility, historical colonial relationships, religiosity (see Boucher, 2018).

The rest of the paper is organised into three main sections. First, it introduces the skill-selective immigration policies deployed by the destination countries. Then, it provides a brief review of the theoretical mechanisms through which immigrants are positively or negatively selected relative to the receiving country’s population. Second, it discusses the theoretical models used in the literature that includes the role of selective policy in their formalisation. Third, it presents the empirical results from different selected studies. There is a final concluding section, which briefly reviews the main findings and future avenues of work.

## Immigration, policy and skill selectivity

A large part of the policy debate discusses how receiving countries may improve their ability to screen potential immigrants. To assess the overall impact of immigration, destination countries care about *who* the immigrants are relative to natives and earlier immigrants, such as whether the immigrant population is composed of skilled or unskilled workers. During the last decade, high-income countries have adopted selective-immigration policies with the idea that keeping out low-skilled immigrants in favour of skilled immigrants may “protect” the host country’s labour markets and welfare systems and address its domestic needs^1^ (Macaluso 2020). There is general agreement among politicians and scholars that higher-skilled migrants are potentially more successful in host-country labour markets. An inflow of high-educated individuals could indeed positively contribute to the economy by bringing valuable skills, stimulating investments, promoting innovation, and raising productivity (see Bertoli et al., 2012). Immigrants are said to be positively/negatively selected concerning relevant observed characteristics, such as higher levels of education, ability to speak the destination country’s language, and income. However, much of the self-selection into migration is explained by several unobserved characteristics—ability, innate talent, motivation, propensity to take risks, which remain difficult-to-observe attributes related to labour market productivity (Borjas, 1987; Chiswick, 1978, 1986, 1999; Gould and Moav, 2016; Bertoli et al., 2016; Borjas et al., 2019). There is a large body of literature in social sciences that shows how migration patterns are embedded in structural differences, social networks, pre-existing cultural similarities and historical ties (e.g. Massey, 1990; Portes and Sensenbrenner, 1993; Hadler, 2006; Polavieja et al., 2018). The selectivity of migration may be influenced by inequality, gender, identities (e.g. ethnicity, class, sexuality), and is inextricably bound up with events and experiences in life-course trajectories, such as family dynamics, housing and retirement plans (see Special Issue edited by Bailey & Mulder, 2017). In this sense, institutional and regulatory changes are crucial in determining the composition of global migration (Donato & Gabaccia, 2015; Green & Hogarth 2017).

Skilled immigrants, who rationally decide to maximise their lifetime utility, are expected to be more beneficial for the host country’s economy because they: (i) pay higher taxes, (ii) require fewer social services, and (iii) integrate faster than unskilled immigrants (Borjas, 2000; Constant & Zimmermann, 2013). Moreover, an inflow of skilled immigrants may have favourable effects on the distribution of income because an increase in the supply of skilled workers will constrain their earnings growth and consequently reduce income inequality, whereas unskilled native workers will be in shorter supply and therefore will have higher wages (Tani, 2014). On the other hand, an inflow of low-skilled immigrants that reduces low-skilled wages would increase income inequality and exacerbate the income gap between high-skilled workers and those less educated (Borjas et al., 1997; Bansak et al., 2015). Despite the concurrent rise in the number of high-skilled immigrants worldwide and the proliferation of selectivity immigration policies, the degree to which high-skilled immigration policies have been effective remains contested (Bhagwati & Hanson, 2009). Responses to these crucial challenges require evidence on the economic mechanisms of migrant selection and policy analyses about the potential selection strategies.

### How quality-immigration policies work at destination

**Fig. 2:**
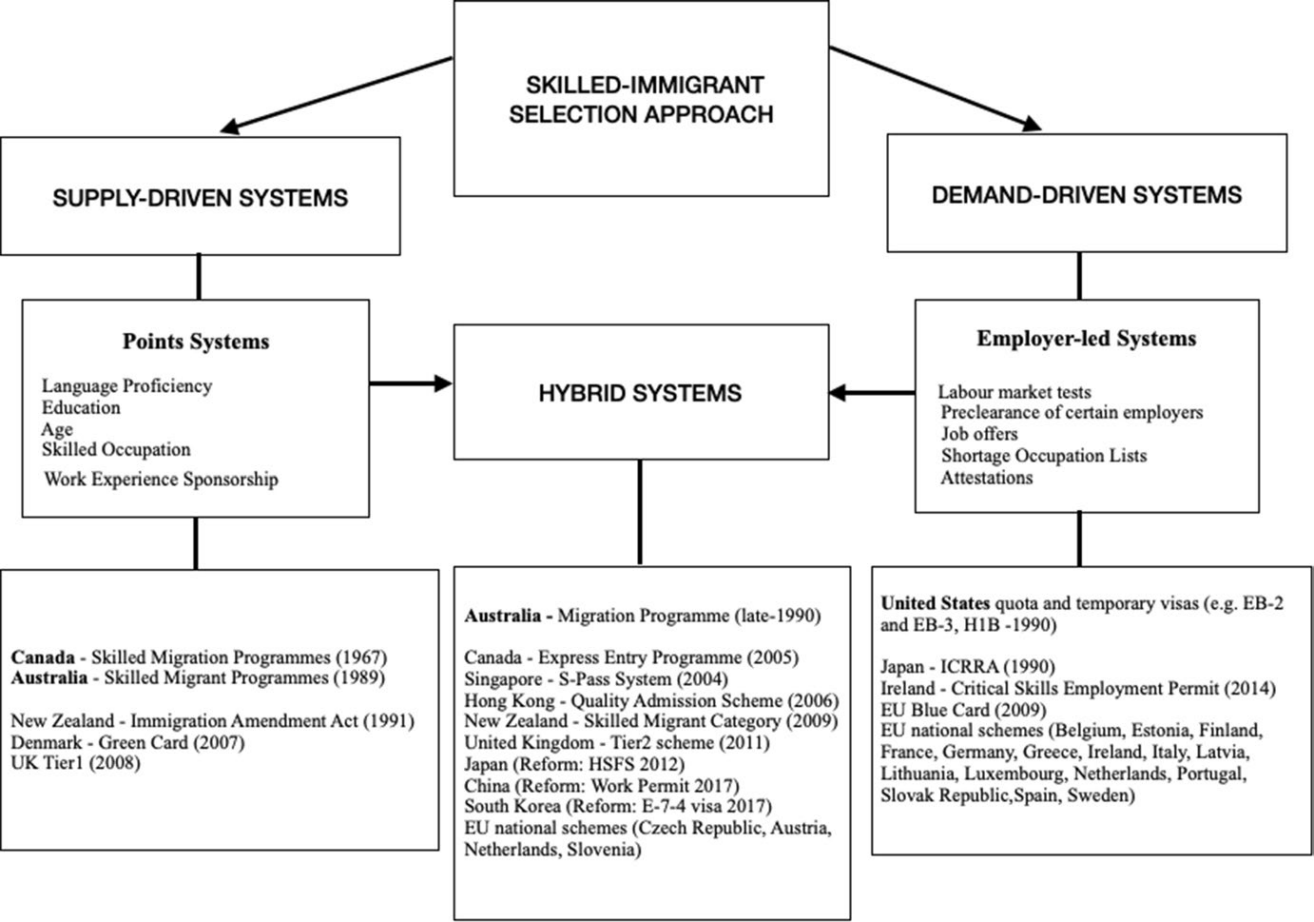
Skill-selective immigration policies in selected destination countries

There are substantial differences across receiving countries in selecting skill-based immigrants. Traditionally, there are two main approaches for skill-based admissions: supply-led systems and demand-driven policies. A third approach is rising recently to meet the need to select immigrants for their education and skills as well as for their ability to fill specific labour market’s needs (Papademetriou et al., 2008; Aydemir, 2020). Figure 2 summarises the skill-selective immigration policies adopted by some selected destination countries.

#### Suppy-led policies

Supply-led policies, like traditional points-based systems, aim at increasing the skill composition of international labour flows by selecting applicants endowed with more desirable individual characteristics of productivity, i.e., educational attainment and language proficiency (Tani, 2014; Bertoli et al., 2016). Points systems focus, in fact, on a government-led selection of high levels of “human capital” in the domestic economy in order to promote long-term economic growth and competitiveness. This argument is typically based on endogenous growth models that emphasise the importance of human capital, knowledge, research and development for economic growth and labour market conditions (e.g. Romer, 1986). Immigrants themselves apply directly to the admission process and pass it based on their education, abilities, and potential for successful integration. Traditionally, points systems have been implemented in countries such as Canada (1967), Australia (1989), and New Zealand (1991), where the initial screening of potential applicants is based on controlling their skill distribution. These countries rank or prioritise applicants based on their desirable individual characteristics, such as level of education, language skills and work experience. If potential immigrants earn enough points, they receive a permanent or a temporary visa.

#### Demand-driven policies

Demand-driven systems, by contrast, allow employers to hire foreign workers on the “need” for migrants’ skills to reduce perceived specific labour and/or skill shortages. Unlike the points-based system, these employer-led programmes are usually based on the principle of job contingency and are often supplemented by a labour market test or, alternatively, they are based on shortage lists assessments. Typically, a demand-driven policy involves the presence of an employment offer and a contract that triggers the decision to admit foreign workers. The government usually determines the cap on total admissions and the eligible occupations (Aydemir, 2020). The US represents the pioneering model for demand-driven policies used for employment-based admissions. Several countries have modified their admission systems to ensure a better selection and a more successful filling of labour/skill gaps, including countries in Europe, Japan, and South Korea (OECD, 2019). The European Union (EU) Blue Card adopted in 2009 is a residence and work permit with elements of a demand-driven policy that requires a valid work contract or a binding offer for a skilled job. With the introduction of the Blue Card, the EU aims to compete globally in attracting high-skilled workers in a context of more and more high international competition (Macaluso, 2020). Other countries have addressed this issue by establishing special government units and/or independent advisory bodies tasked to help analyse shortages in the domestic labour market. For instance, the UK established the Migration Advisory Committee (MAC) in 2008 to advise the government on migration issues, especially in the areas of skilled labour shortages (OECD, 2014).

#### Hybrid policies

Recognising that neither selection system could meet all the needs of selective migration, several governments have shifted their policies towards hybrid systems (Papademetriou et al., 2008; Papademetriou & Sumption, 2011; OECD, 2019). The ultimate goal is to attract highly skilled workers into their countries while meeting employers’ labour needs. Although considering the points system as the most important entry route for highly skilled permanent immigration, Canada and Australia have increasingly recognised the advantages of employer selection and have adjusted their systems to rely on it more heavily. In this respect, the point system based on a few socio-economic characteristics—i.e., education, age and English language proficiency—awards also points to workers in needed occupations. Several other countries, including the UK, Czech Republic, Denmark, Hong Kong, Japan, Singapore, Sweden, have recently introduced a point system to screen economically desirable immigrants depending on a job offer and skills shortages. The US also admits immigrants with “extraordinary ability” (EB1 visa) without any job offers or labour certification (Aydemir, 2020). Different developed countries are facilitating the transition into the labour markets for international students graduating from host country education institutions. Attracting international students and retaining them once they complete their degrees is considered an important gateway to increase the number of needed highly skilled workers (Bertoli et al., 2012; Beine et al., 2014) as well as to maintain domestic institutions’ competitiveness and help to offset some of the effects of demographic decline in developed nations (Hawthorne, 2008). Student migrants are expected to experience a positive selection even though their utility maximisation should be primarily addressed towards skills’ accumulation and then to long-term labour market success (Kogan, 2011).^2^

### The selection of immigrants: a theoretical model

In most economic models of migration decision, cross-country differences in economic conditions play a central role in determining whether people become immigrants (Smith, 1776; Hicks, 1932; Sjaastad, 1962). Migration decision is traditionally modelled as an investment (Sjaastad, 1962), where migrants calculate the value of opportunity in the market at each alternative destination relative to the country of origin, net of the costs of moving, and choose the destination which maximises the highest expected income.^3^ Because migration is not a random phenomenon, self-selection is likely to result in a group of migrants whose personal characteristics are distributed differently from that of the whole origin and destination country populations due to many factors that determine whether people decide to migrate or stay in the home country (see Van den Berg & Bodvarsson, 2009; Cattaneo, 2009). The basic framework used in models of skill selection is related to the Roy model of occupational choice, which investigated how the nature of self-selection into occupations affects the distribution of income so that the allocation of skills in the occupations provides distinct returns depending on the abilities of the workers (Roy, 1951).^4^ This model was, then, introduced to the literature on international migration in a series of influential papers by Borjas (1987, 1991, 1999a) to answer the question “Should I stay or should I go”, such as to determine which persons find it most worthwhile to migrate abroad. Roy/Borjas model considers income inequality across countries as a key indicator to determine the self-selection of workers with different skills. Assuming that migration is an irreversible decision, people decide whether to migrate based on earnings opportunities abroad (${w_{1}}$) and at home (${w_{0}}$). In this framework, potential log wages are determined by an observed component (${\mu _{i}}$, where ${i = 0}$ indicates origin and ${i = 1}$ indicates destination country) and an unobserved component (${\epsilon _{i}}$). Considering *C* be migration costs and $\pi$ be a ’time equivalent’ measure of migration costs given by $C(migration costs)/\mu$ (wages), migration decision is expressed by the index function *I*,

1 $$\begin{aligned} I = log (w_1 / (w_0 + C)) \approx (\mu _1 - \mu _0 -\pi ) +(\epsilon _1-\epsilon _0) \end{aligned}$$

where people migrate if $I > 0$, such as when their wage gains, net of migration costs, are larger in the destination than in the home country. Specifically, how immigrants differ from non-migrants and where immigrants are likely to be in the distribution of wages are driven by cross-countries returns to skills, which, in turn, are related to the extent of income inequality measured by the variance in wages in both countries (${\sigma _{0}}$ and ${\sigma _{1}}$).^5^ This theoretical prediction relies on the correlation coefficient between earnings in both the origin and destination country, $\rho =\displaystyle \frac{\sigma _{01}}{\sigma _0\sigma _1}$ ($\sigma _{01}$ is the covariance ${\sigma _0, \sigma _1}$), which is a measure of the “transferability” of skills (Borjas, 1987). When $\rho$ is positive—skills are transferable—and inequality is higher in the destination than in the origin country, high-skilled workers want to migrate to take the opportunity to earn higher returns in the destination country, and a *positive selection* occurs. Conversely, a *negative selection* occurs when income inequality is higher in the origin than in the destination. In this case, less-skilled workers, which are penalised due to a high wage dispersion, want to migrate to take advantage of a more equal distribution of wage abroad (Borjas, 1987)^6^. These predictions assume that migration costs are constant so that all individuals require the same number of labour hours in order to migrate and therefore do not affect the nature of self-selection (Borjas, 1987, 2014). However, both explicit pecuniary and implicit and psychological costs play an important role in migration decisions. Later works by Borjas (Borjas, 1991; Borjas et al., 2019) and other authors (see Chiswick, 1999; Trübswetter & Brücker, 2004; Chiquiar & Hanson, 2005; Brücker & Defoort, 2009; McKenzie & Rapoport, 2010; Grogger and Hanson, 2011; Gould and Moav, 2016) consider the costs to vary across members of the immigrant pool randomly or correlate to skill levels.^7^ Depending on the size of migration costs and the shape of the skill distribution, migrants may be negatively or positively selected in terms of skills. Chiswick (1999), for instance, shows that positive selection occurs the greater the effect of ability on lowering the costs of migration and the smaller the relative skill differentials in the lower-wage origin relative to the destination. Grogger and Hanson (2011) propose an alternative model where positive selection occurs when the wage difference between the source and destination, net of skill-varying migration costs, is greater for high-skill workers^8^. When migration costs depend on skills, some studies find also an *intermediate selection*, which occurs when immigrants are from the middle of the skill and wage distribution (see, Chiquiar & Hanson, 2005; Orrenius & Zavodny, 2005; McKenzie & Rapoport, 2010). Chiquiar and Hanson (2005) and McKenzie and Rapoport (2010), for instance, develop a model where time-equivalent migration costs decrease with educational attainment ($\delta _\pi s$, where $\delta >0$ is the return to schooling and *s* is the level of schooling), such as ($ln (\pi ) = \mu _0\pi - \delta _\pi s$), and the direction of selection depends on the range of schooling distribution (see also Cuecuecha, 2005). In addition, McKenzie and Rapoport (2010) allow for network effects in the selection process, so that the effect of introducing or expanding networks (*n*) is to decrease migration costs (*C*) at all schooling levels ($C = C(n), C' < 0)$. Therefore, the possible outcomes of both positive and negative selection depend on how the migration decision is modelled and empirical assessed.^9^

## How to formalise a selective-policy in the selection models

Migration literature has paid attention to modelling the role of public policy in determining whether people become immigrants (see among others Greenwood and McDowell, 1991; Clark et al., 2007; Mayda, 2010), and the impact of selective immigration policies on migrants’ education and welfare in the sending country (see, Stark & Wang, 2002; Beine et al., 2008; Bertoli and Brücker, 2011). For instance, selective immigration policies set by destination countries are considered, especially in the literature on the ‘brain drain’ (see, Mountford, 1997; Beine et al., 2001; Bertoli & Brücker, 2011), as a mechanism that acts on the educational attainments and productivity in the origin country. However, the relationship between quality-selective immigration policy and immigrants’ skill composition has been relatively neglected in the formalisations, primarily because admission policies are hard to disentangle from the effects of other characteristics of the host country (Ortega & Peri, 2009; Bertoli et al., 2011).

Immigration policies in the destination countries play a critical role in influencing the direction of selection. However, the Roy model typically ignores the fact that migration flows occur within a policy framework where some receiving countries enact detailed restrictions specifying which potential migrants are admissible and which are not (Borjas et al., 2019). Considering that self-selection may work on how different potential migrants respond to a policy change, some authors have modelled the influence of immigration policies in terms of costs that immigrants have to pay, implicitly or explicitly, to enter the receiving country (e.g. Bellettini & Ceroni, 2007; Giordani & Ruta, 2013; Bianchi, 2013). Migration costs are, at least partly, induced by the host countries, which use a variety of admission policies to limit the number of immigrants entering their countries and shape the characteristics of the inflows. For Grogger and Hanson (2011), the impact of destination-country immigration policies, which influences migration costs, may depend on migrant’s skills due to time costs associated with migration or skill-specific immigration policies in the destination. Specifically, the policy is considered as a mechanism contributing to shaping the pattern of migration costs (Belot & Hatton, 2012) or a mechanism that determines the eventual design of migrants’ self-selection jointly with other determinants of migration, such as the presence of networks (Bertoli & Rapoport, 2015). When migration costs affect the characteristics of their immigrant inflows, their variations will influence the quality of immigrants, as they alter the intensity of selection (see Bertoli et al., 2016). The following paragraph describes the main theoretical models that account for the presence of selective-immigration policies in their formalisation.

### Theoretical frameworks

Self-selection is central to understanding how a policy influences the skill composition of the migration flow. Belot and Hatton (2012), for instance, use a variant of the Roy model to estimate the determinants of educational selectivity, including immigration policy. In this framework, selective immigration systems act as a screening mechanism that imposes differential costs on potential immigrants by skill and education and influences the composition of immigrants rather than the total number. Therefore, immigration policy raises the costs of migration, such that the policy cost (*P*) for individual *i* is $P_i = \delta _0 - \delta _1s_i$, where $s_i$ is the individual education level, which ranges from 0 to 1 (they assign the value 1 to the high-educated and 0 to the low-educated). If policy is not skill-selective, then $\delta _1 = 0$. A toughening in policy raises the policy cost of immigration by increasing $\delta _0$, while an increase in skill-selectivity, holding overall toughness constant, can be achieved by increasing both $\delta _0$ and $\delta _1$. As the Roy Model predicts, a positive selection is given by an increase in the return to skill at the destination relative to the origin country. Further, positive selection is related to direct migration costs and policy selectivity term $\delta _1$ as well as to the degree of poverty and cultural distance.

According to Bianchi (2013), restrictions may improve or worsen immigrants’ skill composition depending on whether immigration is driven by incentives or wealth constraints and on whether returns to skills are higher in the sending or the destination country. In this case, restrictions only affect the migration cost, and they act unconditionally on skills. Skill composition (*Q*) is defined as the ratio of high (${q_H}$) to low (${q_L}$) skilled migrants ($Q =\frac{q_H}{q_L}$). In this case, positive selection occurs if and only if $Q \ge 1$, such as when the number of high-educated migrants is larger than the number of low-educated migrants, something that does not just depend on the returns to education (as in Roy/Borjas model), but also the composition by education of the population in the migrant-sending country. In this framework, policy acts on the composition effect by changing the average skill of immigrants. Therefore, higher restrictions increase the skill ratio if and only if they increase immigrants’ skill composition (*Q*). Instead, Bellettini and Ceroni (2007) extend the assumption of positive selection (as in Chiswick (1999) where skilled immigrants are more efficient in migration), finding a negative relationship between the wage level and the percentage of educated workers among immigrants. For this reason, they argue in favour of higher immigration quotas, which would increase immigrant quality and maximise national income by reducing wages in the receiving country.

Conversely, Bertoli and Rapoport (2015) find that a greater openness of immigration policies increases the expected return to the investment in education. They propose a discrete-choice model where heterogeneous individuals, in terms of ability, make their (endogenous) education decisions while considering the costs that are influenced by the size of networks and expected benefits that depend on foreign wages and the probability of admission at the destination. In this framework, positive self-selection occurs if the utility gain from migration increases with education. Migrants’ quality is defined as an increasing function of the ratio between the number of skilled and unskilled migrants and the additional chances to be admitted at a destination for more educated applicants. Therefore, the quality of migrants and migration networks can be positively associated when destination countries adopt sufficiently selective immigration policies only if the increase in the size of networks induces an increase in the threshold level of ability. Further, they derive two main predictions. First, when policies are non-selective, the relationship between migrants’ quality and networks size is negative (see also McKenzie & Rapoport, 2010; Beine and Özden, 2011). Second, the presence of quality-selective immigration can enhance the quality of migrants in the long run because they can rise to a positive relationship between migrants’ quality and the size of networks. Also, in Bertoli et al. (2016), selective policies act on migrants’ quality on the cost side. Following the literature (e.g. Borjas, 1987; Aydemir, 2011), they define the quality as the average log wage that both high-educated and low-educated immigrants earn at the destination, and it is maximised when the probability of self-selection for skilled immigrants is greater than that for unskilled immigrants. This is, in turn, influenced by the variation in education-specific migration costs since they act by modifying the intensity of selection on unobservables. Migration costs are considered, at least partly, policy-induced by the host country through the legal framework that regulates immigrants’ admission at destination and is higher for uneducated potential migrants. A change in selective-immigration policy affects the quality of immigrants in two ways. First, if selectivity increases, the share of skilled immigrants increases as well. Second, it changes the intensity of selection for the two groups of migrants since the quality-maximizing share of skilled immigrants is a decreasing function of the scale of migration.^10^ The extensions of their basic model show that the prevailing pattern of selection on unobservables can negatively affect migrant quality when migrants have, on average, a higher level of ability than stayers. Therefore, selective immigration policies might fail to achieve their primary goal of raising migrant quality, although they increase the share of educated immigrants.

## Empirical studies on skill-based policies and migrants’ selection

According to Borjas (1999b, p.180), the analysis of the immigration policies should start from three related questions concerning: (1) the nature of the tradeoff among the well-being of natives, (2) the well being of immigrants, and (3) the well-being of the population in the origin country. It is often argued that stricter immigration policies have a positive selection effect on immigration from the host country’s perspective, as it makes migration less attractive for less-skilled individuals. As pointed out by Bertoli et al. (2012), immigration laws that favour high-skilled immigrants relative to less educated ones are among the most important and robust correlate affecting the positive selection. Moreover, they find that laws limiting immigrants’ access to welfare benefits and increasing the restrictions for residence permits produce a small total inflow of immigrants and a more substantial selection bias of the immigrant population towards the highly educated (Macaluso, 2020). However, some negative factors emerge from introducing a selective immigration policy. Policies that screen applicants by education are probably the best to protect the economic well-being of the native population. Still, it may negatively affect the welfare of other groups of people, e.g. the origin country may lose its best individuals (Borjas, 1999b; Speciale, 2010).

A strand of the literature investigates the role of immigration policy on the socio-economic performance of immigrants in the destination country, especially by empirically looking at a causal impact, and the US context (Kerr & Lincoln, 2010; Kato & Sparber, 2013; Pope, 2016; Shih, 2016; Mayda et al., 2018; Steigleder & Sparber, 2017; Clemens et al., 2018; Chassamboulli & Peri, 2020). However, these papers do not directly investigate the relationship between the composition of flows and the type of admission policy. Instead, others empirically investigate the question of how countries should attract the most skilled immigrant flow, also prompting by some evidence over the decline in selection on education levels of immigrant waves in some traditional destination countries (see Borjas, 1995; Aydemir & Skuterud, 2005; Borjas & Friedberg, 2009).

Most studies have devoted their attention to Canada and Australia that implemented for long periods systems to directly screen immigrants according to their individual characteristics. Other studies have compared Canada and Australia’s points systems to the US’s admission policies, which differ notably in labour market policies and institutions. Theoretical selection models (as in Borjas, 1987) predict that generous redistribution systems and relatively more equal wage structures as in Australia and Canada should attract less-skilled immigrants from the lower end of the income distribution (Antecol et al., 2003). However, most of the evidence underlines that immigrant selection based on skill requirements —e.g., point-based systems in Canada and Australia— may be effective in receiving a more skilled immigrant flow than the US. Evidence on other countries remains sparse. This literature usually compares different measures of observable skills —language fluency, education (years of schooling), and income— in the destination countries. However, other (more recent) papers emphasise the role of unobserved characteristics in determining the quality and relevance of human capital immigrants. Focusing only on observed characteristics may indeed result in skill transferability problems or mismatches between the demand for specific skills in the host country and their supply through immigration (see Bertoli et al., 2016; Borjas et al., 2019). This issue may affect how host countries can benefit from immigrant selection mechanisms.

Available evidence on selection outcomes by visa category is mainly hampered by data limitations concerning the flows, the type of immigrants, and the destination policy. First, most available data combines all kinds of migrants and do not discriminate for economic migrants, refugees and family-based immigrants. Then, a few databases include variables capturing the selectivity dimensions of immigration policies. The existing databases with information on visa categories do not provide any measure of skill, except in some cases, occupation. Specifically, most available datasets do not distinguish between immigrants admitted via employment or skills criteria and those coming through other channels. Another limitation concerns the presence of policy settings that are becoming more and more complicated. This section reviews the existing empirical studies on the effects of a selective immigration policy on migrants’ composition and quality in the destination countries.

### Empirical evidence: Canada and Australia vs the US

Several analyses rely on the traditional points-based systems in Canada and Australia, often compared to the visa-preference system used in the US. Comparative studies investigate how these receiving countries differ in their policies and attribute migrants’ outcomes to the different admission policies. Table 1 reports some selected empirical studies based on a single receiving country or a comparative study, taking into account the reference periods in the implementation of the selective systems, the data and the measure of skills, and the main results in terms of patterns of selection.

**Table 1:** Selected studies from traditional quality-selective systems

Study	Country	Data	Time span	Measure of skills	Results
Green and Green (1995)	Canada	Quarterly dataset: cansim and occupational data	1955–1993	Education occupation	Positive effect of skill-selective admission policies on composition of new immigrants
Cobb-Clark (2003)	Australia	Longitudinal Survey of Immigrants to Australia (LSIA)	1993–1995 1999–2000	Education English ability	Positive selection based on increased emphasis on skills
Aydemir (2011)	Canada	Longitudinal Survey of Immigrants to Canada (LSIC)	2000–2001	Education Language ability	Positive selection based on skill requirements
Jasso et al. (2000)	US	New Immigrant Survey Pilot (NIS-P)	1992–1996	Education	Immigrants admitted under employment-based visas are more educated than other immigrants
Bertoli and Stillman (2019)	US	American Community Survey (ACS)	2001–2017	Education, unobservables	A further increase in the selectivity on education is unlikely to improve substantially the quality of migrants coming to the US
Tani (2020)	Australia	Survey (LSIA)	1960–1981	Education labour market outcomes	Selective-migration policies increase quality of immigrants but no significant impact on labour market outomes upon arrival
Borjas (1993)	Canada US	Census	1960–1981	Educational attainment wages	Skill filters are effective because they alter the allocation of visas across source countries
Antecol et al. (2003)	Australia Canada US	Census	1990–1991	Language fluency education income	Australian and Canadian immigrants have higher levels of English fluency,education and income than do US immigrants
Jasso and Rosenzweig (2008)	Australia US	Survey: LSIA2, NIS	1996–2000	World prices of skills country proximity	No evidence that differences in the selection mechanism play a significant role in affecting the characteristics of skill migration

Antecol et al. (2003) find, as expected, that Australian and Canadian immigrants are positively selected in terms of education, English fluency and income than do US immigrants. However, when they exclude immigrants from Latin America, the observable skills are similar in the three countries, suggesting that geographic and historical ties play a more critical role than skill-based admissions. This is consistent with Borjas (1993) who find that immigrants in Canada have about a year more schooling at the time of arrival and a wage advantage (relative to natives) than immigrants in the US (for the role of the selection process in generating immigrant outcomes in Canada and the US see also Chiswick, 1987; Duleep & Regets, 1992, 1996; Friedberg, 2000; Warman et al., 2015). However, the average skill level of specific national-origin groups is about the same in Canada and the US. Thus, point systems work better not in attracting more skilled workers from a particular country but because it alters the national-origin mix of the immigrant flow. Also, Jasso et al. (2000) find that the relationship between immigrants’ educational attainments and visa category depends mainly on the origin country so that immigrants from different countries differ in the routes by which they qualify for a visa (see also Mattoo et al., 2008). In this respect, immigrants who qualified under the employment-based systems in the US —especially screened for skills in high demand— tend to have the highest levels of schooling relative to immigrants admitted via other categories.

Contrary to Borjas (1993) and Jasso et al. (2000), Aydemir (2011) shows that point systems select more skilled immigrants within countries of origin rather than by changing the country of origin composition. Hence, the Canadian selection system based on skill requirements effectively increases the average level of migrants’ education and language abilities. However, positive selection in human capital characteristics does not necessarily imply better labour market outcomes. According to Green and Green (1995), the introduction of a point-based system in Canada (1967) —which made skills selection more stringent— accounts for shifting the immigrant composition from low to high skilled in the short run. Instead, only significant policy shifts significantly affected the composition of immigration in the long run. Further, the effect of skill-selective policies become lower over time due to the increasing role of family-network dynamics. Although the estimation includes the national origin mix of immigrants over different periods, this analysis does not identify the individual effects of the policy from the changes in incentives to migrate. Looking at immigrants selected based on their employment or skill characteristics, Jasso and Rosenzweig (2008) find that Australia attracts higher-skilled migrants than does the US. In this case, differences in the selection rules employed by the two countries explain immigrant self-selection more in terms of country’s proximity and skill price, which in Australia is lower than the US, so that immigration gains are more significant from immigrating to the US. However, this analysis does not find any evidence concerning the role of the two different selection mechanisms in shaping the characteristics of skill migration. Conversely, Cobb-Clark (2003) shows that policy changes, which emphasise productive skills in the selection process, led individuals entering Australia (the late 1990s) to have both better education and language skills than individuals entering five years earlier.^11^ Further, the effects of changing human capital endowments are not uniform but concentrated amongst those most likely to be subject to tighter selection criteria. However, Miller (1999) finds that the variations in immigrant quality in Australia are likely to be affected more by conditions in the worldwide market for skilled immigrants than by the Australian points system. Further, Tani (2020) confirms that the 1990 policy change in Australia raised the quality of affected migrants (as in Cobb-Clark, 2003), but had no significant impact on indicators measuring immigrants’ skills, suggesting that itself, may not be able to address issues related to the labour market.^12^ In addition, a recent paper by Bertoli and Stillman (2019) finds that point systems, which heavily rely on education, might fail to improve the quality of immigrants markedly. Consistent with the theoretical model in Bertoli et al. (2016), the reason can be traced to the effect that these systems have on the pattern of selection on unobservables, which can play a more critical role than the direct effect of selection on observables embedded in the policy. Therefore, most of the variance in log wages for US immigrants may be explained by the differences in unobservables within rather than across countries.

Overall, evidence on Australia and Canada highlights that skill-based immigrants have higher human capital endowments than migrants selected by other policies. Furthermore, immigrants entering under systems that emphasise skills —i.e., education and the ability to speak the host’s country language— experience a more positive selection among immigrants. However, several authors have stressed that skill-based systems have very little ability to affect immigrants’ skills and long-term success in the receiving country. Moreover, the selected studies do not consider the recent changes in the skill-selective immigration systems, especially in Canada and Australia. Whereas, few studies have investigated the role of the US demand-driven system in selecting immigrants.

### Empirical evidence on other Countries

While some papers have investigated the question of whether immigration restrictions affect selection (see Bertoli et al., 2011; Grogger & Hanson, 2011; Borjas et al., 2019), there has so far been little analysis examining the effects of selective migration policies on ’non-traditional’ systems. Table 2 summarises the results obtained by these studies, which are mainly cross-country comparisons.

Belot and Hatton (2012) investigate the effects of skill-selective immigration policies in 21 OECD destination countries by using three indicators: (1) responses of business to a question on how immigration policy allows the hiring of foreign employees, (2) restrictiveness of the country’s policy towards professional workers, and (3) a dummy for countries that select immigrants through a points-based system. Here, the non-selective effect is captured by the share of time when the analysed countries allowed free labour mobility. Results highlight a positive selection related to points systems and policies that screen immigrants on skills. Instead, some adverse effects for welfare magnet selection emerge, particularly after controlling for other policies. However, these measures capture elements of post-immigration policy that shape migration incentives. Conversely, the lack of indicators that measure the potentially significant effects of policies —e.g. family reunifications and refugees— do not allow to obtain the overall impact of policy on skill selection.

**Table 2:** Selected studies from ’non-traditional’ selective systems

Study	Country	Data	Time span	Measure of skills	Main results
Constant and Zimmermann (2005)	Germany Denmark	Rockwool Foundation Migration Survey	1984–2002	Education	Labour migrants are more educated than those under other entry schemes
Belot and Hatton (2012)	OECD countries	OECD-based dataset	1980–2001	Education	Positive selection based on points systems and policies that encourage the transfer of professional skills
Docquier and Rapoport (2007)	EU15 vs other OECD countries	Dataset by Docquier and Marfouk (2006)	1990–2000	Education	Selective-policy have only a moderate impact on selection of immigrants than other determinants
Bertoli et al. (2012)	OECD countries	Panel on Ortega and Peri (2009)	1980–2000	Education	Selective policies are among the most important and robust correlates which affect the positive selection of immigrants
Bertoli and Rapoport (2015)	OECD countries	Dataset by Brücker et al. (2013)	1980–2010	Education	An increase in the size of migration networks leads to a worsening in migrants’ quality in nearly all non-selective destinations

Few papers have devoted their attention to the role of skill-selective policies in shaping the quality of immigrants, especially in the EU context, and concerning other determinants of selection. Bertoli and Rapoport (2015), for instance, show that the adoption of selective immigration policies could be influencing the relationship between networks and quality of immigrants and can have lasting effects on the education structure and skill composition of immigration. Specifically, an increase in the size of migration networks does not systematically change the quality of immigrants so that quality-selective immigration policies could be effective in the long run. Analyses on the impact of the Blue Card Directive have mainly focused on the number of high-skilled workers who arrived under this scheme and the effects in some EU labour markets. Still, they do not inform about the mechanisms of immigrant selection. Constant and Zimmermann (2005), for instance, highlight that immigrants who arrived in Germany and Denmark under a work permit are more educated than those who came under other schemes (family reunification and refugee/asylum status). Thus, they advocate the introduction of a selective immigration policy to address the demands of the labour market in the EU (see also Ruhs and Anderson, 2010). However, Docquier and Rapoport (2007) highlight that a change in immigration policy that increases the degree of selection could have only a moderate effect on the composition of flows. Further, a selective process could help mitigate some demographic and economic trends. Still, it may be limited because (i) the EU suffers from some structural problems, as a considerable skill and labour shortage compared with other immigration countries, and (ii) a selective European policy could increase regional inequalities. Other studies, instead, show that the EU could benefit from a migration policy in line with economic needs. Czaika and de Haas (2017) find evidence that supply-led systems, like points-based systems, aim to increase both the absolute numbers of high-skilled migrants and skill composition of international labour flows. Moreover, Czaika and Parsons (2017) present a cross-country assessment of policies aimed to attract and select high-skilled workers. This analysis shows that points-based systems are much more effective in attracting and selecting high-skilled migrants than demand-driven policies, which have a slight effect —and potentially even a negative one.

In sum, while some evidence on ‘non-traditional’ selective countries suggests that imposing selection requirements based on skills raise immigrants’ average quality, other studies tend to find only a moderate effect on the composition of flows. However, most of these papers are devoted more to assessing skill-based admission policies’ effectiveness than to empirically considering their role in immigrant selectivity across countries.

## Conclusion

This paper presented a survey of the most theoretical and empirical analyses of the relationship between selective immigration policies and the composition of incoming immigrants’ inflows from an economic perspective.

During the last decades, screening for immigrants with specific socio-economic characteristics or labour demand needs has become increasingly common among developed countries. However, there has been little analysis on the role of skill-selective policies in the selection process from the receiving country perspective. Evidence is limited and mostly related to the traditional countries of immigration, such as Australia, Canada and the US.

Most of these studies show that immigrants selected based on observable characteristics —education, age, occupational composition and ability to speak the destination country’s language— are more positively selected than do other immigrants, especially in Canada and Australia (Borjas, 1993; Cobb-Clark, 2000; Antecol et al., 2003; Cobb-Clark, 2003; Jasso & Rosenzweig 2008; Aydemir 2011). This is not consistent with the canonical selection model, which predicts that countries with higher income inequality (e.g. the US) are expected to attract the most educated individuals from countries offering relatively low rates of return to skills. Conversely, countries with a more compressed income distribution (e.g. Australia and Canada) will attract less-skilled immigrants from the lower end of the income distribution. However, the Roy Model does not consider that skill-selective policies influence the direction of selection, admitting only certain groups, or lowering migration costs for more educated immigrants. Therefore, it can expect that point-based systems that screen for workers based on desirable observed characteristics improve migrants’ quality among immigrants, particularly for a country with a relatively high average income but with a compressed wage structure (Antecol et al. 2003; Tani 2014). From a theoretical point of view, migration costs are shaped, at least partly, by the policy in the host country, which regulates immigrants’ admission and is higher for uneducated potential migrants. Therefore, higher restrictions act on the skill ratio if they increase immigrants’ skill composition (Bianchi, 2013), or immigrants’ quality (Bellettini & Ceroni, 2007), and when increases the threshold level of ability associated with an increase in the size of networks (Bertoli & Rapoport, 2015). However, although they increase the share of educated immigrants, selective immigration policies might fail to achieve their primary goal of raising migrant quality (Bertoli et al., 2016). Some studies do not confirm indeed that policies that screen migrants based on observable characteristics are better off in attracting the immigrants from the top part of the skill distribution (Bertoli & Stillman, 2019). Much of the selection is embedded in the unobserved characteristics —ability, innate talent, motivation, propensity to take risks. So, it can be expected that migrants will be self-selected also in terms of unobserved characteristics since migrants’ selection is responsive to policy regimes at the destination. Therefore, an important question is related to the role played by unobservable characteristics because it helps better assess the effects on the quality of migrants and how self-selection will reflect the relative skill prices. This issue raises questions about the effectiveness of point systems that are necessarily based on observable characteristics and the costs and benefits of international migration, including the ongoing debates on brain drains and gains. Further, other studies find that skill-selective policies act in altering the national-origin mix (Borjas, 1993; Green & Green, 1995), rather than attracting more skilled workers from a particular source country (Aydemir 2011). Evidence on employer-driven schemes is less clear cut. Some studies find that immigrants admitted via employment-led systems tend to be more educated than other immigrants Jasso et al. (2000), others instead find minor effects or even harmful in attracting highly skilled immigrants Czaika and Parsons (2017). However, these studies stressed that skill-based systems had very little ability to affect immigrants’ skills and long-term success in the receiving country (e.g. Miller, 1999; Cobb-Clark, 2003; Jasso & Rosenzweig, 2008; Tani, 2020). Recent evidence indeed suggests that skill-selective immigration policies can have lasting effects on the education structure and skill composition of immigration (Bertoli & Rapoport, 2015). Future research should make more effort in quantifying the impact of the admission policy choices by capturing their composition effect both in the theoretical and empirical designs.

There are other promising avenues of research. One relevant question relates to the role of the selective policies in shaping cultural factors and social makers, i.e. gender inequality, family ties, ethnic enclaves, marriage and fertility, historical colonial relationships, religiosity, which are largely understudied in the literature on migrants’ selection (Rapoport et al., 2020; Docquier et al., 2020). Different studies have suggested that other determinants of migration —geographical proximity, historical ties, networks, cultural similarities, skill prices— often play a more significant role in the self-selection process than selective immigration policies (Miller, 1999; Antecol et al., 2003; Jasso & Rosenzweig, 2008; Belot and Hatton, 2012). While social networks have been treated as an essential factor in the cost of migrating (Bertoli & Rapoport, 2015), other social makers should also be considered both in theoretical formalisations and empirical frameworks. For instance, gender differentials could significantly impact transnational networks that could facilitate migration and satisfaction around opportunities (Itzigsohn & Giorguli-Saucedo, 2005). Further, they could shape the pool composition of migrants in the country of origin and their perception of the cost of migrating (Massey, 1990; Curran & Saguy, 2001). Some of these factors could remain difficult-to-observe attributes by policymakers but are likely to have a substantial impact in defining migration quality.

Whether immigrants screened for skills are better selected than those selected via other visa categories is hampered by the presence of complicated policy settings. Furthermore, suitable data largely ignore the specificities of differentiated groups of migrants such as humanitarian, unskilled and skilled, and international students. While there is increasing evidence on selectivity among migrant populations at the aggregate level, overall selectivity patterns among skilled and educated migrants are not well-understood. How countries set different immigration policies is crucial in determining the size and the composition of flows, which determines the economic impact of immigration both at destination and in the origin countries. Whether the adoption of selective immigration policy in the destination countries may produce a change in the quality of immigrants and how a change in the selection policies, e.g. a tightness in the admission policies, is likely to affect the direction and the magnitude of immigrant selection, remain still open questions, which deserve more attention from researchers. From a policy perspective, policymakers should consider these results and be aware of both the effects and (unintended) consequences their policies may generate. An important question could address how the impact of new shocks, e.g. Brexit or the Covid-19 pandemic, has put under pressure the “traditional” selective systems.

## Footnotes

1 The definition of ’skill’ is debated both in academic studies and among domestic governments (Boucher, 2020). The economic literature often uses education as a proxy for measuring the skill level, which is usually defined on the basis of wages paid (Docquier & Marfouk, 2006; Chaloff & Lemaitre, 2009; Bansak et al., 2015). Therefore, a ’high-skilled migrant’ is typically one with at least tertiary education (university degree), who belongs to the upper part of the skills distribution (in terms of education and work experience) in both the origin and the destination countries (Czaika, 2018).

2 National policies and high education institutions in destination countries are highly interested in supporting inflows of foreign students and improving their stay rates, especially in countries that have deployed significant efforts to the internationalisation of students and researchers (UNESCO, 2019; OECD, 2020). There is a huge literature on migration and development concerning the role of public institutions in shaping migration of international students (see e.g. Borjas, 2005, 2009; Dustmann and Glitz, 2011; Kato & Sparber, 2013), especially related to the brain drain and brain gain phenomena (see among others Beine et al., 2008; Rosenzweig, 2008; Clemens, 2009; Spilimbergo, 2009; Docquier & Rapoport, 2012; Beine et al., 2014). However, this point encompasses the purpose of this paper.

3 The view of migration as a human capital investment stemmed from Becker (1962), which hypothesised that people invest in their skills to maximise the net present value of future earnings.

4 The Roy (1951) multiple-index model focuses on how the nature of selection in the occupational choice influences the distribution of income. Three factors affect the choice of workers’ selection: (1) distribution of skills and abilities and their correlations; (2) technologies for applying these skills; (3) consumer tastes that impact the demand for different types of outputs. Then, workers self-select the sector that gives them the highest expected earnings.

5 Scholars use different ways to measure the return to skill. The most used are: (i) the Gini index; (ii) income shares and income ratios; (iii) comparisons among earnings of workers in different skill groups.

6 Borjas (1987) shows a third case of selection—*Inverse sorting* or *refugee sorting*—that occurs when immigrants are from the low end of the wage distribution in the origin country while being in the upper end of the distribution at the destination. In this case, the correlation between earnings in the two countries is sufficiently low (Borjas, 1987, 2014). This is the case, for example, of the refugees. However, because refugee migration is not motivated by potential economic reasons, the direction of selection depends on the nature of the refugee-producing event and other idiosyncratic factors (Chin & Cortes, 2015).

7 Borjas (1991); Borjas et al. (2019) extend the model to include stochastic migration costs, so that $\pi$ varies across individuals. However, the results confirm the findings of the basic model as long as the migration costs are unrelated to potential earnings.

8 The prediction of “generalized” positive selection (Borjas, 2014, p.23) is criticised by Borjas (Borjas, 1987, 1991, 1999a), highlighting that immigrants who self-select in response to skill premium, observed distributions of skills across the source and destination countries’ populations, and perceptions of their own skills, cannot earn high incomes in the destination country.

9 Empirically, most of the existing literature examines the selection of immigrants according to levels of education, with a large share dedicated to the study of Mexican immigrants to the US (see among others Chiquiar & Hanson, 2005; Orrenius and Zavodny, 2005; Ibarraran & Lubotsky, 2007; McKenzie & Rapoport, 2010; Ambrosini & Peri, 2012; Moraga, 2013; Kaestner & Malamud, 2014). Few papers investigate the self-selection of high-skilled migrants towards the developed countries (Grogger & Hanson, 2015; Parey et al., 2017), especially considering also the role of the unobserved characteristics involved in the migration decision (Gould and Moav, 2016; Borjas et al., 2019).

10 Bertoli et al. (2016) confirm these two main prepositions also by extending their basic specification to four implications. Specifically, they extend their framework to allow for (i) a more significant variance in the log wages of educated immigrants; (ii) a random variation in time-equivalent migration costs; (iii) an alternative information structure following Bertoli (2010) in contrast with Borjas (1987, 1999a) by considering wages not remotely observable; (iv) considering selective policy as a change in the size of the information set upon which educated agents to take in the decision to migrate.

11 Evidence on the role of immigration policy on the performance of immigrants in their destination countries and how immigration impacts these countries’ economies and native populations highlight the importance of skill and geographic composition of immigrant cohorts and how it can be affected through policy tools.

12 Specifically, Tani (2020) refers to the use of the immigrants’ skills in the labour market outcomes upon arrival, including the issues of overeducation (when actual education level is higher than that required to perform their job tasks) and occupational downgrading (when occupation after the migration is of a lower level than that of the last job performed before migrating (see Dustmann et al., 2016).
